# Polyene-Based Derivatives with Antifungal Activities

**DOI:** 10.3390/pharmaceutics16081065

**Published:** 2024-08-14

**Authors:** Kwanele Ngece, Thabisa L. Ntondini, Vuyolwethu Khwaza, Athandwe M. Paca, Blessing A. Aderibigbe

**Affiliations:** Department of Chemistry, University of Fort Hare, Alice 5700, Eastern Cape, South Africa; 201102901@ufh.ac.za (K.N.); 201709075@ufh.ac.za (T.L.N.); apaca@ufh.ac.za (A.M.P.)

**Keywords:** polyenes, antifungal activity, potency, hybrid compounds, polyene-based derivatives

## Abstract

Polyenes are a class of organic compounds well known for their potent antifungal properties. They are effective due to their ability to target and disrupt fungal cell membranes by binding to ergosterol and forming pores. Despite their effectiveness as antifungal drugs, polyenes have several limitations, such as high toxicity to the host cell and poor solubility in water. This has prompted ongoing research to develop safer and more efficient derivatives to overcome such limitations while enhancing their antifungal activity. In this review article, we present a thorough analysis of polyene derivatives, their structural modifications, and their influence on their therapeutic effects against various fungal strains. Key studies are discussed, illustrating how structural modifications have led to improved antifungal properties. By evaluating the latest advancements in the synthesis of polyene derivatives, we highlight that incorporating amide linkers at the carboxylic moiety of polyene molecules notably improves their antifungal properties, as evidenced by derivatives **4**, **5**, **6G**, and **18**. This review can help in the design and development of novel polyene-based compounds with potent antifungal activities.

## 1. Introduction

Multidrug resistance (MDR) is a serious complication in the treatment of diseases, including fungal infections [1]. Over the past several decades, fungal pathogens have emerged as significant contributors to human morbidity and mortality, with *Candida* species reigning as leading casual agents of life-threatening invasive fungal infections [2]. Such diseases occur primarily as complications of surgery, chemotherapy, hematopoietic and/or solid organ transplant, or immunosuppressive therapies [3], making antifungals critical to the success of these modern medical advances [2]. Invasive fungal diseases account for 1.6 million deaths per year worldwide [4]. Multidrug resistance (MDR) in fungal pathogens has been increasing over the last decade. Importantly, among other mechanisms, such as drug mutations, efflux-based MDR has been recognized as a major cause of fungal anti-infective drug resistance. The small number of chemical entities in antifungal drugs has been very problematic, especially after prolonged use, as was the case for pronounced azole resistance in HIV patients [1]. Underlying host conditions, antifungal pharmacokinetics and pharmacodynamics, and fungal attributes may alone or collectively contribute to therapeutic failure [5]. Fungal failures resulting in antifungal resistance involve various subcellular mechanisms, including alteration of drug target, overexpression of efflux pumps and drug target, and gross chromosomal changes [6]. It is important to highlight the difference between tolerance and resistance. Antifungal tolerance involves acute cellular responses to stressors that threaten the integrity of the fungal cells, such as antifungals. Antifungal resistance is either acquired [7] or innate (inherent) and is defined when a species intrinsically exhibits elevated MIC values toward an antifungal [8].

**Figure 1:**
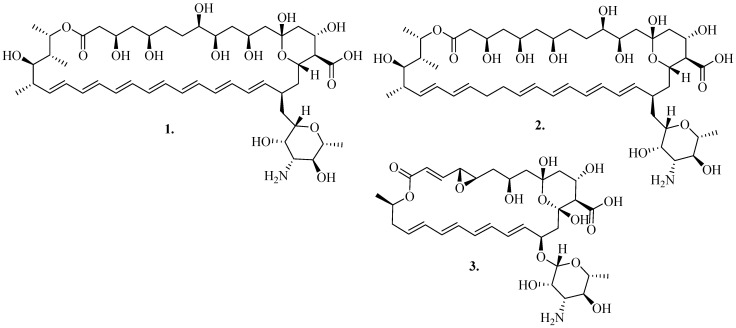
Chemical structures of polyene antibiotics: **AmB**(**1**), **nystatin A1**(**2**), and **natamycin**(**3**).

Fungal infections such as *Candidosis*, *Aspergillosis*, and *Cryptococcosis* are responsible for causing clinical infections in individuals with weakened immune systems [9]. *Candida albicans* is another fungal pathogen that opportunistically thrives in patients with weakened immune systems and is linked to numerous cases of mortality globally. The virulence factors linked to the pathogenicity mechanism of *C. albicans* include morphological changes, the development of biofilms, and the release of hydrolytic enzymes [10]. There are notable variations in Candida’s susceptibility to various antifungal drugs. Polyene antifungal drugs are a class of antimicrobial compounds known for their potential effect in treating fungal infections. Over two hundred polyene compounds have been discovered and shown to be the first antifungal antibiotics. Among them are amphotericin B (AmB), nystatin, and natamycin (Figure 1), which are frequently administered to treat fungal infections [11]. Polyenes continue to be administered due to their broad antifungal effects against harmful yeasts and molds, including various species such as *Aspergillus* spp., *Candida* spp., *Cryptococcus* spp., *Rhizopus* spp., *Fusarium* spp., etc. [12].

These polyenes are extensively used in treating systemic fungal infections, despite their notable toxicity and the necessity for parenteral administration owing to their modest absorption from the gastrointestinal tract [8]. Their chemical structures (Figure 1) consist of a hydrophobic heptane chain and a hydroxyl-rich hydrophilic chain, which gives the molecules an amphiphilic feature, resulting in minimal solubility in aqueous solutions. This limited solubility has prompted the development of numerous formulations of polyenes aimed at mitigating the complications linked to their intravenous administration, such as renal toxicity [13]. It is essential to develop an efficient and simple method for synthesizing a variety of biologically effective molecules. Developing synthetic drug molecules tailored for organic purposes represents the primary pathway for advancing drug innovation. In recent years, research laboratories worldwide have concentrated on developing new reactions and reagents to discover medically potent drug analogues. The synthesis of polyene-based derivatives involves various methods, including structural modifications of the parent polyene structure, structural optimization through medicinal chemistry approaches, and the development of novel synthetic routes [12,13]. Recent advancements in synthetic methodologies have enabled the generation of diverse polyene derivatives with enhanced pharmacological properties and reduced toxicity profiles. Structure–activity relationship (SAR) studies have elucidated key structural features responsible for the biological activity of polyene derivatives. Rational design strategies guided by SAR principles have facilitated the development of novel derivatives with improved therapeutic profiles [14]. In this review, we present polyene derivatives, their structural modifications, and the influence on their therapeutic effects against various fungal strains.

## 2. Fungal Infections

As eukaryotic organisms, fungi exhibit remarkable variation in appearance and lifestyle, enabling them to potentially colonize vast regions [15]. There are two main types of fungi: mold and yeast. The majority of yeast cells are small, oval, single cells, whereas the mold colonies are formed of filamentous strands referred to as hyphae. Certain fungi are dimorphic, meaning that they can exist as molds or yeasts depending on the surrounding conditions (such as temperature, etc.) [16]. In contrast to systemic fungal infections, which can affect several organs, such as the brain, skin, kidneys, liver, etc., local fungal infections can affect any part of the body [17]. A widely reported cause of high morbidity and mortality is the rising incidence of fungal infections in immunodeficient patients, including the elderly, those receiving cancer therapy or organ transplants, COVID-19 patients, and those with implanted medical devices [18]. There are over 120,000 known species of fungi [19]. Fungal infections caused by *Aspergillus*, *Cryptococcus*, *Pneumocystis*, *Mucormycosis*, and *Candida* species are responsible for 1.5–2 million deaths [20] and affect over a billion people worldwide [21]. The most prevalent fungal infection in the orofacial region is *Candidiasis*, which is followed by *Aspergillosis* and *Mucormycosis* [22]. *Candidiasis* and *Aspergillus* species cause extreme redness, itching repercussions in skin tissue, swelling, bloody urine, and pelvic pain [23].

Candida species have the potential to infect the oral cavity as both commensal and invasive pathogens [24]. *C. albicans* [25] is an opportunistic pathogen that causes *Candidiasis*, especially among immunodeficient patients [26]. Non-albicans *Candida* spp. poses significant risks to human health [27] and has surfaced worldwide due to growing rates of antifungal resistance [28]. White-to-grey lesions and a burning feeling inside the mouth constitute a few of its symptoms. This infection is exacerbated by *Candida* spp.’s virulence factors, such as morphogenesis, adhesions, and biofilm formation [29]. Mucormycetes are members of the class Zygomycetes and the causative agents of Mucormycosis, also known as Zygomycosis, responsible for high morbidity and mortality rates [30]. The most frequent saprophytic environmental fungi that cause infections are *Rhizomucor*, *Aposphysomyces*, *Saksenaea*, *Cunninghamella*, *Rhizpous*, and *Licitheimia* [31]. Mortality rates can range from 10% to 100%, depending on the infection site and underlying risk factors [32]. A potentially fatal systemic mycosis, *Cryptococcosis,* is commonly seen in immunocompromised people, primarily in HIV-positive patients. The two main basidiomycetes yeasts that induce cryptococcosis are *Cryptococcus neoformans* and *Cryptococcus gattii* [33]. The fungus is extensively dispensed in the environment and is typically linked to the droppings of pigeons and different types of trees, such as eucalyptus. Spores or dehydrated fungal cells can be swallowed by the host to cause infection. Alveolar macrophages phagocytose fungal cells once they enter the lungs; nevertheless, in immunocompromised individuals, fungal cells can evade the intrinsic immune system’s barrier and spread all over the body [34]. *Aspergillus* is a taxonomic classification for conidial fungi or asexually growing fungi. *Aspergillus* members can be classified as Ascomycota members, pending the availability of DNA evidence, since a few of them are recognized to exhibit teleomorphism (the sexual state) within Ascomycota [35]. Significant mortality rates of 90% from invasive aspergillosis have been reported [36]. Air-bone spores from the common environmental mold *Aspergillus fumigatus* are often inhaled. The fungus is a strategic pathogen, and although healthy people’s immune systems are very good at removing infectious spores from their lungs, patients with weak immune systems are at risk of developing invasive pulmonary aspergillosis, a lung disease that progresses quickly and is frequently fatal [37]. Non-fumigatus *Aspergillus* spp., inclusive of *A. flavus*, *A. niger*, *A. terreus*, *A. parasiticus*, *A. ochraceus*, are to be taken seriously [38]. The rising usage of antibiotics contributes to the evolution and spread of AMR, a serious and urgent public health crisis.

## 3. Antifungal Activity of Polyenes

One of the most powerful classes of compounds in the battle against fungal infections is polyenes. They develop in the molecular channels of lipid and cell membranes that are selectively permeable to ions and chemical substances [39]. They have lactone rings in their molecules. Moreover, even though all polyenes possess potent properties against fungal diseases, a few of them are not potent [40]. Six polyene antifungals—nystatin, **AmB**, natamycin, methyl partricin, candicidin, and trichomycin—have been used as antifungal therapies. Among the six polyenes, only three are commonly used in antifungal therapies: natamycin for ocular infections, **AmB** for integral invasive fungal infections, and nystatin for mucosal infections (oral infections or vulvovaginal candidiasis) [41]. Despite extensive research, the mechanism of action of polyenes is still not fully understood. The earliest reported mode of action was the development of pores following binding to sterols found in the cell membrane [42]. Sterols play a significant role in the permeability and plasticity of eukaryotic membranes, but they are uncommon in bacteria. In addition, sterols from eukaryotes have been observed to generate functional membrane microdomains in bacteria [43]. Polyenes such as amphotericin B exert their antifungal effects through a mechanism that disrupts fungal cell membranes. Specifically, polyenes bind to ergosterol, a key component of fungal cell membranes, forming pores or channels within the membrane. These pores alter the permeability of the membrane, leading to the leakage of essential cellular components and ions, ultimately causing fungal cell death. This mechanism is selective to fungal strains because while fungal cell membranes contain ergosterol, mammalian cell membranes contain cholesterol instead. This selectivity reduces the likelihood of adverse effects on human cells, making polyenes an effective treatment for fungal infections. However, despite their efficacy, polyenes also have side effects due to their potential to bind to mammalian cell membranes at high doses, leading to toxicity.

Targeting the ergosterols is essential for all antifungal drugs. Ergosterol, a crucial component of fungal cell activity, is responsible for cellular processes, such as cell signaling, endocytosis, cell division, membrane fluidity, and membrane protein control [41]. One of the primary concerns among researchers and physicians is the increasing AMR against currently available antifungal medicines. Resistance to existing therapeutic drugs has made treating pathogenic fungi, viruses, bacteria, and protozoa challenging [44]. Multidrug-resistant microbial infections can cause difficulty in patients undergoing chemotherapy, surgery, and transplantation. These infections can include nosocomial, skin structure, and urinary tract infections [45]. The search for novel antifungal drugs like polyene derivatives has increased due to the mechanisms that lead to drug-resistant fungal cells and the evolution of drug-resistant infections.

### 3.1. Polyenes and Their Derivatives

#### 3.1.1. Amphotericin B (AmB)

**AmB** (Compound **1** in Figure 1) is a polyene used to treat systemic mycotic infections. It is produced by *Streptomyces nodosus* [46]. Due to its broad-spectrum antifungal efficacy, it is commonly used. **AmB** destroys harmful fungi through a variety of molecular processes [47]. It favourably binds to ergosterol, forming trans-membrane channels that promote the intracellular leakage of K^+^ and Mg^2+^ ions and nutrients, causing cell death. **AmB** is a two-edged sword that has a broad antifungal spectrum, good therapeutic impact, and reduced drug resistance; on the other hand, it is very toxic to mammalian cells, causing hepatotoxicity, hemolytic toxicity, and nephrotoxicity [48]. Due to the severity of its side effects, research has been conducted to synthesize **AmB** derivatives to reduce the side effects while retaining their potency against fungal pathogens. Volmer et al. documented numerous **AmB** derivatives with potential biological effects developed by 2010. Their review highlighted a significant increase in the range of structural modifications [49].

**Figure 2:**
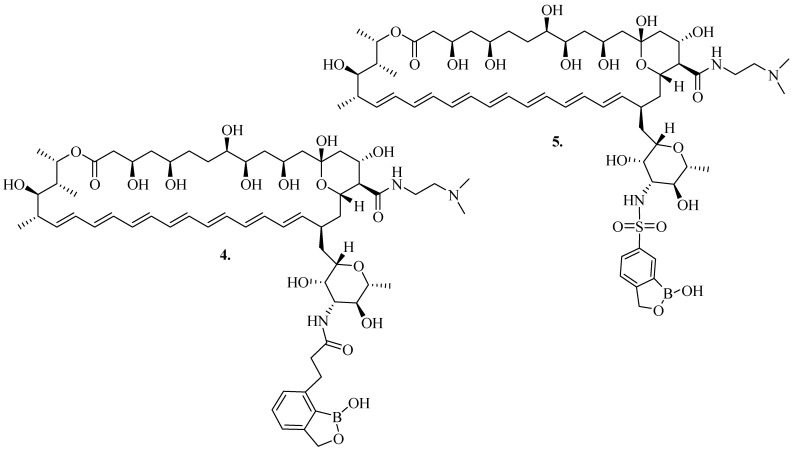
Structure of dual modified derivative C16-DMAE-amide (compounds **4** and **5**).

Tevyashova et al. formulated innovative series of derivatives of **AmB** conjugated with benzoxaborole moieties and evaluated their antifungal potency against selected fungal strains *C. albicans*, *Cryptococcus humicolus*, *Aspergillus niger*, and *Fusarium oxysporum* [50]. Most of the novel compounds were reported to be less effective than **AmB**. Notably, dual altered derivatives such as C16-DMAE-amide compounds **4** and **5** (Figure 2) displayed significant antifungal efficacy (MICs = 0.25–1 µg/mL) when compared to **AmB** (~0.25–2 µg/mL) against *A. niger*, *C. albicans*, *C. humicolus*. The introduction of 3′-*N*-[3-(1-hydroxy-1,3-dihydrobenzo[c][1,2]oxaborol-7-yl)propanoyl] (**4**) and 3′-*N*-(1-hydroxy-1,3-dihydrobenzo[c][1,2]oxaborol-6-yl)sulfonyl (**5**) to **AmB** led to more enhanced antifungal activity against the aforementioned strains.

Notably, the hybrid benzoxaborole-**AmB** antibiotic compounds **4** and **5** were similarly potent as the parent **AmB** against *C. albicans*, *C. humicolus,* and *A. niger*. However, compound **5** was toxic to mammalian cells, making this derivative disadvantageous. Thus, the introduction of the benzoxaborole residue resulted in increased antifungal activity of compound **5**; however, associated with increased cytotoxicity in comparison with **AmB**. In compound **4**, a potent antifungal activity was paralleled by low toxicity for human cells and hemolytic activity better than **AmB**. Compound **4** demonstrated optimal characteristics in all three tests, that is, antifungal activity, cytotoxicity and hemolytic activity. This agent was further prepared as a water-soluble, pharmaceutically acceptable l-Glu salt **4G** potentially useful in animal models [50].

**Figure 3:**
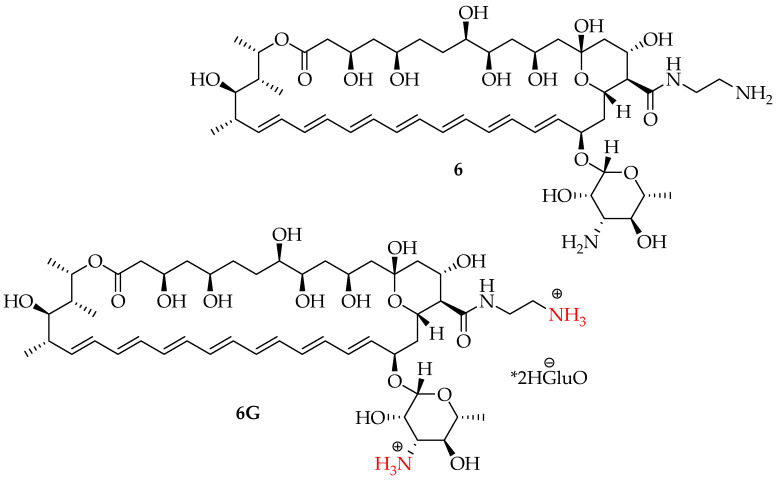
Structure of compound **6** (amphamide) and its salt **6G**.

Tevyashova et al. formulated a series of **AmB** derivatives and tested their antifungal activity against the strains of *Candida* spp. (*C. krusei* 432M, *C. albicans* ATCC 24433, *C. glabrata* 61L, and *C. tropicalis* 3019) and filamentous fungi (T. *rubrum* 2002 and *M. canis* B200) and compared it to that of **AmB** [42]. Their study revealed that when tested against 209 strains of filamentous fungus and Candida, the amide derivatives exhibited strong antifungal activity. Compound **6** (Figure 3), also known as amphamide, was found to have greater antifungal activity than **AmB** against filamentous fungus and all the tested *Candida* that were examined (see Table 1). The water solubility of AmB’s N-(2-Aminoethyl) amide is modest. However, its l-glutamate salt (**6G**) (Figure 3) was found to be soluble and stable in water. Furthermore, compound **6G** exhibited in vitro antifungal potency comparable to that of compound **6**’s free base form. Therefore, it was determined that the amidation of AmB’s C16-carboxylic group with ethylenediamine (compound **6**) was advantageous in boosting the antifungal activity and enhancing its solubility in an aqueous medium. Amphamide, in contrast to **AmB**, can be utilized as glutamate **6G**, a water-soluble salt, greatly simplifying the formulation and administration of dosage medications. As a result, the synthetic amphamide offers significant chemotherapeutic benefits over **AmB**. This therapeutic candidate could facilitate the development of the next generation of polyene antibiotics for the treatment of fungal infections [42].

The electrophysiological experiments of the selected candidate **6** suggested that it had lower self-aggregation properties but higher pore-forming abilities in the model membrane compared to those of **AmB** under the same conditions. The enhanced selectivity of amphamide (**6**) for the ERG-containing bilayers resulted in a lower toxicity of compound **6** compared to that of **AmB**. In vivo studies in the model of mice candidosis sepsis confirmed that compound **6** had much lower acute toxicity and higher antifungal efficiency. Thus, a novel **AmB** derivative, amphamide (**6**), with considerably increased safety and better efficacy compared to those of **AmB** and with a therapeutic index (LD_50_/ED_50_) almost 17-fold higher than that of **AmB** (42 vs. 2.4), was discovered. Moreover, unlike **AmB**, amphamide (**6**) can be used in a water-soluble salt form, which considerably simplifies dosage drug formulation and application. Thus, the synthesized amphamide has substantial chemotherapeutic advantages over **AmB**.

Gurudevan et al. synthesized **AmB**–albumin conjugates (**BSA-AmB**) and evaluated their antifungal susceptibility against selected fungal strains, using **AmBisome** as a control [51]. The MIC values for free **AmB** against the three strains ranged from 0.4 to 0.8 µg/mL. It is interesting to note that the conjugate’s **AmB** MIC values were marginally higher than the free **AmB**, ranging from 0.6 to 1 µg/mL as demonstrated in Table 2. However, the MIC value of **AmBisome** was between 0.78 and 1.5 µg/mL, which is higher than the values obtained for the **BSA-AmB** conjugate. The amine group (NH_2_) found in **AmB** was useful in forming hydrogen bonds with ergosterol to create pores on fungal membranes, which increased its antifungal activity. Albumin’s numerous binding sites, inherent transport mechanisms, and cellular connections make it a suitable intravenous drug delivery carrier. Conjugating **AmB** with albumin was a viable option to increase **AmB**’s solubility, reduce its toxicity, and enhance its pharmacokinetics [51].

It is noteworthy that **BSA** has shown to be a good solubility enhancer in the case of **AmB**. The drug was found to be in its monomeric form in the conjugate and was not aggregated. A slow and steady release of the drug from the conjugate has a significant role in increasing its half-life when administered systemically. Drug release from the conjugate in human plasma was very similar to the release seen in the case of **AmBisome**, suggesting that the pharmacokinetic profile of the conjugate when administered intravenously would probably be similar to **AmBisome**. Conjugation dramatically mitigated the toxicity and hemolytic activity of **AmB**. The conjugate also exhibited potential antifungal activity comparable to the activity of **AmBisome**.

**Figure 4:**
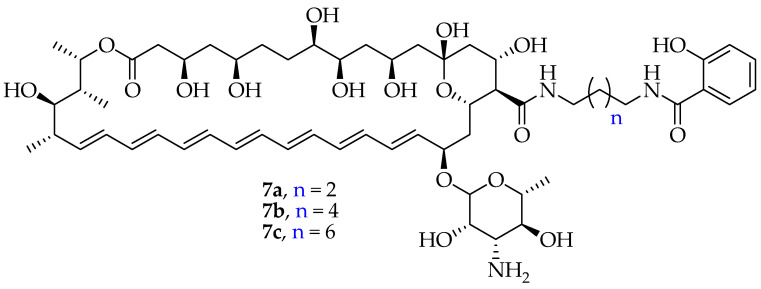
Structure of AmB derivatives containing a salicylic acid moiety.

Yu et al. synthesized **AmB** conjugates (**7a**–**7c**) (Figure 4) containing a salicylic acid moiety and evaluated their antifungal activity against selected *Candida* spp. strains to determine their MICs [48]. The reduced antifungal activity of the conjugated compounds **7a**–**7c** was over 2 to 8 times lower than that of **AmB**. Compound **7a** was characterized by a short two-methylene chain, and **7b** and **7c** contained an increased chain length. However, conjugates **7a**, **7b**, and **7c** displayed immensely low nephrotoxicity, even at high concentrations, with **7c** displaying the lowest hemolytic toxicity and **7b** showing a higher toxicity. Thus, modifying **AmB** by adding a salicylic acid moiety is an efficient approach to sustain **AmB**’s antifungal efficacy while lowering nephrotoxicity [48].

The length of linkage chains has an obvious effect on the antifungal activity of the derivatives. The shortest two-methylene chain derivative, **7a**, exhibited the best antifungal activity. However, the antifungal activity of the derivatives decreased slightly with increasing chain length, whereas both **7b** and **7c** also retained good antifungal activity. The hemolytic toxicity and nephrotoxicity of **AmB-SA** derivatives were significantly reduced compared to those of **AmB** (see Table 3). Compounds **7a**, **7b**, and **7c** showed very low nephrotoxicity, even at high concentrations; **7c** exhibited the lowest hemolytic toxicity and **7b** had higher toxicity than the other two compounds, but still far lower than that of **AmB**. Additionally, the hemolytic toxicity of **7a** was between those of **7b** and **7c**. The promising results were an encouragement to put more effort into the evaluation of their in vivo safety and antifungal activity.

**Figure 5:**
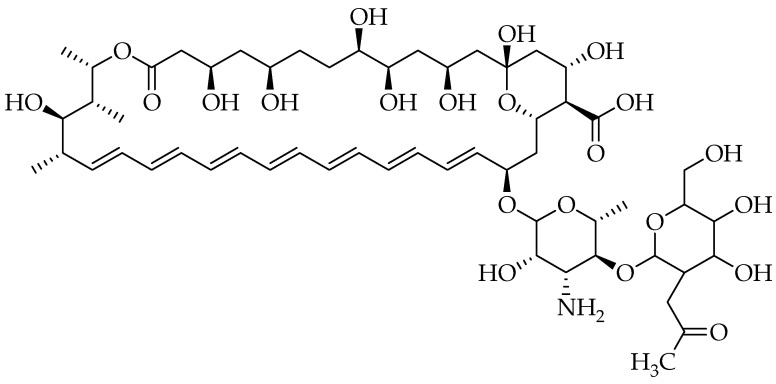
Structure of compound **8**, an **AmB** derivative.

Kim et al. formulated a **nystatin**-like *Pseudonocardia* polyene **8** and evaluated the antifungal activity of the compound (Figure 5) against different fungal strains [52]. Compound **8** displayed a 2-fold lower antifungal potency than **AmB** (see Table 4). Moreover, the hemolytic activity of compound **8** was slightly decreased compared to that of **AmB**. Compound **8** demonstrated a similar antifungal effect against *C. albicans* but had a lower toxicity than **AmB**. The manipulation of the specific polyketide enoyl reductase (ER) domain in the **nystatin**-like *Pseudonocardia* polyene biosynthetic pathway led to compound **8** being a potent antifungal agent. The compound displayed enhanced pharmacokinetic parameters compared to **AmB**, suggesting that compound **8** could be a potential candidate for development into a pharmacokinetically enhanced and less toxic polyene antifungal drug [52].

It is noteworthy that compound **8**, with its unsaturated C28–C29 bond, exhibited significant antifungal activity when compared to other derivatives. Furthermore, compound **8** demonstrated enhanced pharmacokinetics and reduced toxicity compared to the structurally similar heptaene **AmB**. The antifungal mechanism of polyene macrolides is believed to involve simple binding to ergosterol, while binding to cholesterol is responsible for toxicity. Preliminary SPR experiments indicate that **AmB** has a higher affinity for both ergosterol- and cholesterol-containing membranes than compound **8**, which preferentially binds to ergosterol-containing liposomes. This difference in cholesterol binding selectivity may contribute to the differing toxicities of **AmB** and compound **8**, which requires further verification. In summary, compound **8** is a promising heptaene alternative to **AmB** with reduced toxicity. In vivo studies suggest that adding an extra sugar residue to heptaene macrolides could improve their pharmacological properties without significantly reducing antifungal efficacy. This study has made considerable progress in expanding the structural modification of compound **8** to improve toxicity.

Alshahrani et al. conjugated **AmB** to a polyethylene glycol carrier and **ZnO** nanoparticles and reported their antifungal efficacy against *C. neoformans* and *C. albicans* [53]. The PEGylated **ZnO-AmB** inhibited or killed fungal cells at significantly low concentrations in comparison with free **AmB** or ZnO-AMB as shown in Table 5. The higher antifungal efficacy of **AmB** conjugated with **PEG** and **ZnO** nanoparticles was attributed to the hydrophilic polymer, (**PEG**), which improves the colloidal solidity of the conjugates, hindering aggregation, and facilitates the uptake of the formulation via the pores of fungal cell membranes. The hematological values of rats administered with the formulation were retained and were similar to the values of the untreated control rats. The nephrotoxic effect of the formulation was not significant, revealing the need for further in vivo studies [53].

Notably, the PEGylated **ZnO-AmB** retained the same hematological parameters as those in uninjected control rats, in contrast to **ZnO-AmB** and free **AmB**, which changed the hematological parameters over those of control rats. Furthermore, creatinine and BUN levels were significantly lowered in the case of injection with **ZnO-AmB-PEG**, in comparison to amphotericin-induced nephrotoxicity. These results emphasize the possibility of clinical applications of PEGylated **ZnO-AmB** at lower concentrations with minimal nephrotoxicity; however, further study is required to investigate the in vivo efficacy of the developed formulation.

#### 3.1.2. Nystatin

Nystatin (Compound (**2**) in Figure 1) is an anti-microbial drug with fungicidal and fungistatic effects that was first discovered in 1951 and has been used topically to treat oral candidiasis. It is derived from *Streptomyces noursei*, and the gastrointestinal tract absorbs very little of it [54]. Due to its inhibitory impact against a wide range of pathogenic and non-pathogenic fungi and yeast, nystatin has garnered a lot of attention [55]. Topical formulations of nystatin are used for the treatment of oral, esophageal, gastrointestinal, genital, and cutaneous candidosis. Nystatin has been utilized for gastrointestinal prevention and the treatment of corneal infections [56]. The main biological target of nystatin is the plasma membrane of the cells of fungi and protozoa, which affects a variety of processes, such as the development of pores. It is well tolerated with a low risk of hepatotoxicity and few adverse effects (such as nausea and vomiting).

**Figure 6:**
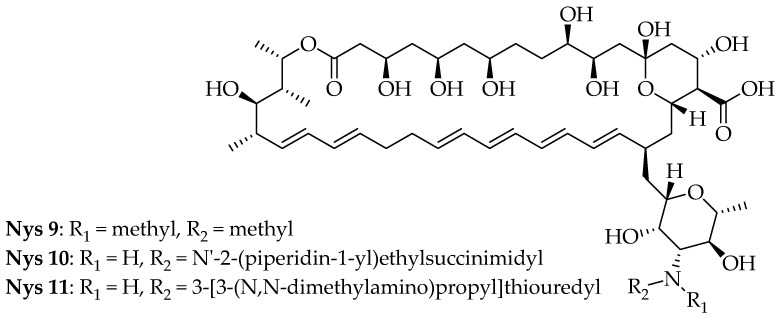
Structures of nystatin derivatives (**Nyt 9–11**).

Boros-Majewska et al. synthesized **nystatin** derivatives displaying low host cell toxicity and promising antifungal activity in an in vitro model of oral candidosis. According to the in vitro antifungal susceptibility experiments, the derivatives were effective against *C. albicans* (ATCC 10231) [57]. Among the derivatives that were evaluated, compound **10** showed a good antifungal effect, with a MIC value of 2 µg/mL, while **9** and **11** showed MIC values of 4 µg/mL (Figure 6). The IC_50_ value of compound **10** was 1.19 µg/mL and it was the most effective antifungal derivative of **nystatin**. It was noteworthy that **9** and **11**, despite their distinct modification types, exhibited similar antifungal activity, with an IC_50_ value of 2.81 µg/mL [57]. The replacement of hydrogen at the primary amine of nystatin by *N*′-2-(piperidin-1-yl)ethylsuccinimidyl is responsible for compound **10** being the most active antifungal. Furthermore, the methyl/methyl (**9**) substitution at the primary amine and 3-[3-*N*,*N*-dimethylamino)propyl]thiouredyl (**11**) at the primary amine of **nystatin** also yielded promising antifungal activities. It is necessary to conduct more research into the mechanism underlying the novel nystatin derivatives’ selective toxicity.

It was particularly interesting to note that the nature of **nystatin** modification appeared to influence the extent of *C. albicans* invasiveness since complete prevention was found only in the case of compound **10** (N-succinimidyl derivative). Additionally, a positive relationship between the MIC (IC_50_) for the derivatives and the extent of *Candida* invasion was detected. It is necessary to mention that these derivatives significantly inhibited *Candida* colonization, including biofilm-like structure formation and highly reduced or completely prevented *Candida* invasion. Additionally, these new polyenes were less toxic against keratinocyte cells and the oral epithelium compared with natural nystatin.

**Figure 7:**
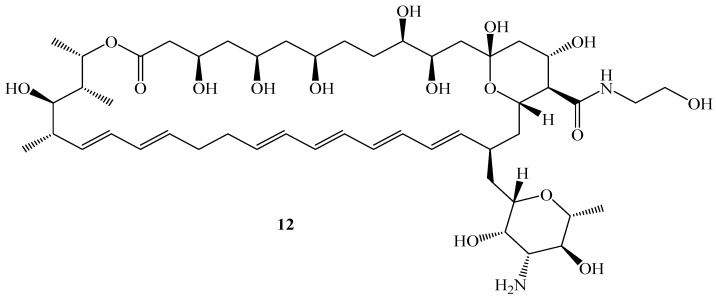
Structure of nystatin derivative **12**.

Amir et al. synthesized nystatin derivative **12** (Figure 7) using primary amines and alcohols. The synthesized hybrid was evaluated for its antifungal activity against two fungal strains (*C. albicans* (ATCC 10231) and *A. niger* (ATC 20057)), in vitro [58]. Notably, compound **12** exhibited promising results against selected fungal strains as shown in Table 6. SAR analysis showed that the amide linker was responsible for the improved antifungal activity, as compound **12** was 13.5 times less toxic than **nystatin**. **Nystatin** derivatives with an amide linker are promising candidates for future drug development, but further studies are recommended for validation [58].

Both **nystatin** and compound **12** are water-soluble and have a potency similar to that of **nystatin**. **Nystatin** is soluble in DMF, whereas compound **12** is soluble in water, which increases its bioavailability without using formulations. In addition, compound **12** was found to be less toxic in vitro than **nystatin**. Compound **12** was stable in human plasma for 2 days with the formation of its isomer. Furthermore, compound **12** at 0.25 mg/mL was found to be 52% bound to human plasma proteins after 24 h.

**Figure 8:**
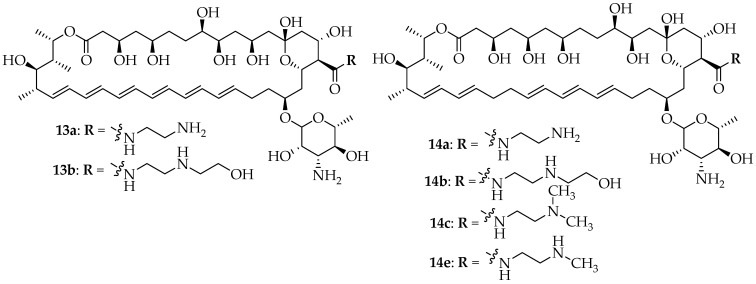
Structures of promising **AmB** (**13**) and nystatin (**14**) derivatives.

Tevyashova et al. synthesized semisynthetic amides of **AmB** and **nystatin A1** and studied their antifungal activity and toxicity ratio [59]. The antifungal activity of polyene derivatives **13a**–**13e** and **14a**–**14e** (Figure 8) was tested against different fungal strains and compared to **AmB** (**13**) and **nystatin** (**14**). According to reports, the novel **nystatin** derivatives exhibited potent antifungal activity that was comparable to **nystatin** (except against *C. krusei* 432M); the amides containing ethylenediamine and *N*-(2-hydroxyethyl)-ethylenediamine compounds **13a** and **13b** showed the most notable findings in the **AmB** group (see Table 7). Additionally, it was observed that, in comparison to **nystatin**, the most active derivative of **nystatin**, **14a**, had three times lower toxicity to kidney cells and displayed comparable hemolytic activity at 10 and 20 µM, with noticeably decreased activity at 50 µM. More active derivatives, **13a** and **14a**, contained an ethylenediamine moiety within the **AmB** and **nystatin** cores, respectively. Additionally, the toxicity of **nystatin** was reduced by the addition of the short aminoethanol group to the **nystatin** molecule. **AmB** derivative **13a** is a promising candidate for prospective drug development. Two water-soluble amides derived from the **nystatin** series, **14c** and **14e**, performed exceptionally in cell toxicity studies. Additionally, it was demonstrated that the most potent **nystatin** derivative, **14a**, was far less toxic than the free drug. As a result, three novel water-soluble **nystatin** compounds were identified as prospective candidates for drug development [59].

**Figure 9:**
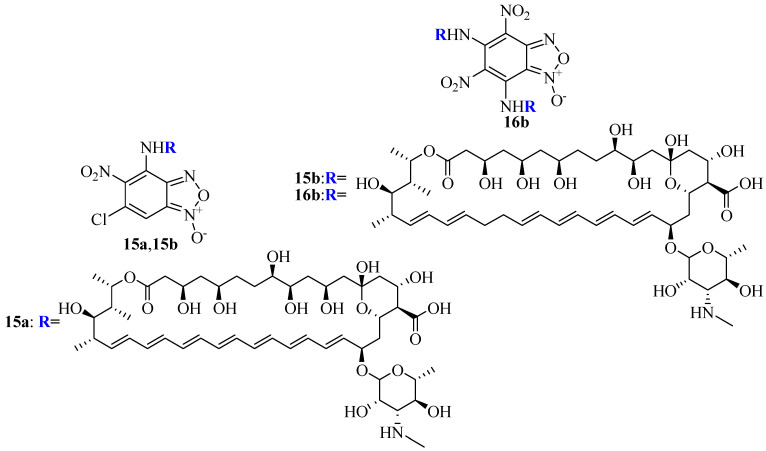
Structures of benzofuroxane hybrids and polyene antibiotics (**AmB** and **Nys**).

Chugunova et al. synthesized hybrid compounds derived from polyene and benzofuroxanes and evaluated their antifungal activity against selected fungal strains [60]. Among the compounds, derivatives **15a**, **15b**, and **16b** (Figure 9) were found to be potent antifungals. The introduction of 4,6-dichloro-5-nitrobenzofuroxans and 5,7-dichloro-4,6-dinitrobenzofuroxans into **AmB** and **nystatin** led to derivatives **15a**, **15b**, and **16b** being the most potent antifungals. Compound (**16b**), prepared from 5,7-dichloro-4,6-dinitrobenzofuroxan and **nystatin**, exhibited four times the activity of **nystatin** against *Trichophyton mentagrophytes*. Fungistatic effects were observed when drugs inhibited the test microorganism’s growth in concentrations not exceeding 500 µg/mL. The lowest concentration of the drug inhibiting the growth and reproduction of the test microorganism cultures was considered the effective dose [60].

#### 3.1.3. Natamycin (Pimaricin)

Natamycin, or pimaricin (Compound **3** in Figure 1), is another potent polyene antibiotic with a long history of use. It is produced by *Streptomyces natalensis* and is applied topically to treat fungal infections. The structure of natamycin is a macrocyclic lactone ring containing four conjugated carbon–carbon double bonds (tetraene) and an amphoteric mycosamine group [61]. Oral and vaginal candidosis can be treated topically with natamycin. Moreover, it has been utilized to treat corneal infections [56]. Natamycin works by attaching itself to ergosterol and interfering with sterol-dependent membrane activities without the need for an ion channel [62]. This mechanism leads to ergosterol-specific and reversible inhibition of membrane transport proteins without changing the permeability of the cell membrane. It also involves the inhibition of the transportation of glucose and amino acids. There have not been any reports on the severe negative effects of natamycin. Rare cases of mild irritability, redness, a burning feeling, stinging, and tears have been reported [63].

**Figure 10:**
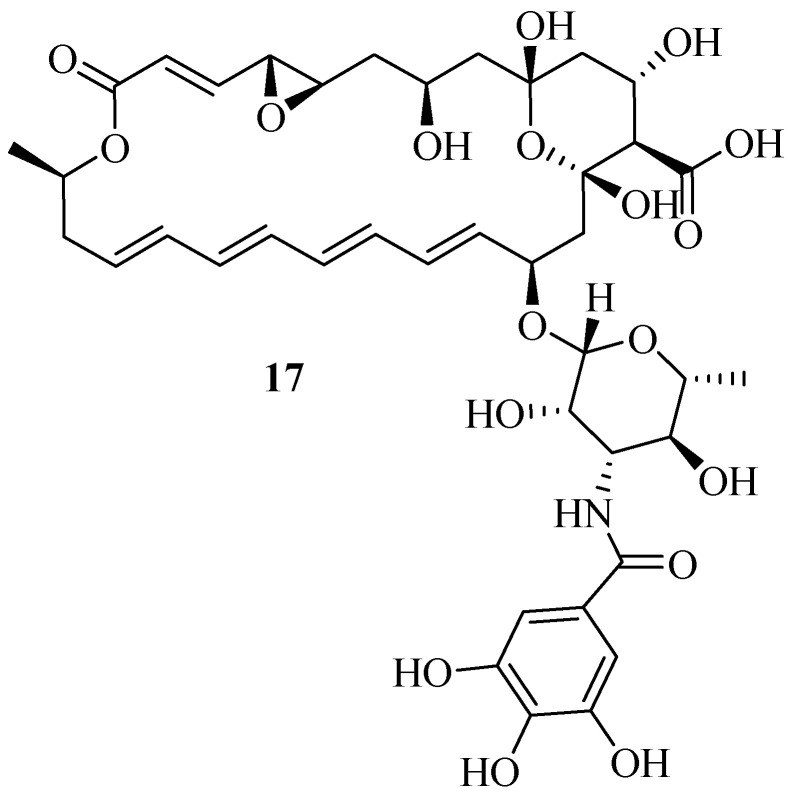
Structure of natamycin derivative compound **17**.

Ji et al. synthesized derivatives of **natamycin** (**NAT**) containing a **gallic acid** (**GA**) moiety to enhance the therapeutic efficacy of **natamycin** on fungal keratitis (FK) [64]. *A. fumigatus* conidia were cultivated for 48 h with varying concentrations of **natamycin** or compound **17** (Figure 10) (2, 4, 8, 16, 32, and 64 µg/mL). At 4 µg/mL, **GA-NAT** significantly reduced *A. fumigatus* growth. Mycelial cell wall staining showed that **GA-NAT** at 8 µg/mL effectively controlled the germination of *A. fumigatus* spores, which showed no difference with **natamycin**. Consequently, **GA-NAT** demonstrated effective antifungal activity at 8 µg/mL. Furthermore, *A. fumigatus* biofilm development was reduced by **GA-NAT** at a dose of 8 µg/mL. **GA-NAT**’s antifungal activity was the same as that of **natamycin** against *A. fumigatus* at the same dose. In addition to suppressing *A. fumigatus* growth, **GA-NAT** also successfully blocked the fungus’s ability to adhere to surfaces and form biofilms, which is a crucial stage in the fungal infection’s resistance to the host immune system. These findings provide more evidence that **GA-NAT** has anti-inflammatory properties and can help shield the cornea from harm by severe inflammation. The addition of **gallic acid**, a well-documented anti-inflammatory [65] and antifungal agent, to **natamycin** has enhanced the efficacy of **GA-NAT** and improved the shortcomings of **natamycin**. This study’s findings demonstrated that **GA-NAT** is less cytotoxic and its antifungal/anti-inflammatory qualities make it a viable treatment for *A. fumigatus* keratitis [64].

Furthermore, in in vivo experiments, **GA-NAT** distinctly inhibited the growth of *A. fumigatus* compared with **natamycin** on the fifth day. Also, through plate counting, the survival of corneal fungal colonies in the **GA-NAT** treatment group was lower than those of the other controls. Thus, it was seen that **GA-NAT** could take a positive role in controlling inflammation and shortening the course of disease in the *A. fumigatus* keratitis model.

**Figure 11:**
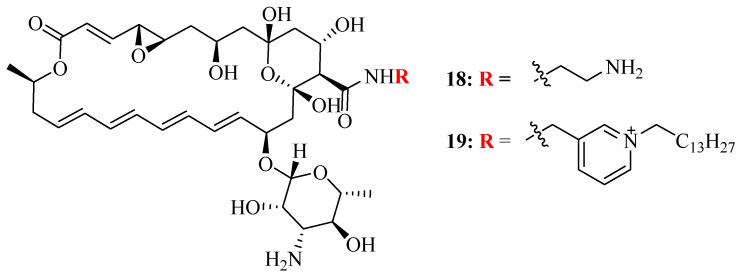
Structure of amide derivatives of **natamycin**.

Tevyashova et al. synthesized a series of semisynthetic amides of polyene antibiotic **natamycin** and evaluated their antifungal activities against *Candida* spp. and filamentous fungi [66]. They reported that amide **18** (Figure 11), with a MIC value of 2 µg/mL, was more potent than **natamycin** (MIC value of 8 µg/mL) against all tested *C. auris* strains. Among the seven derivatives, amide **19,** with long lipophilic side chains, showed the highest efficiency index (EI) and strong antifungal activity in vitro but was more toxic against human postnatal fibroblasts (HPFs). In in vivo experiments, amide **18** showed in vivo efficacy in a mouse candidemia model with a larger LD_50_/ED_50_ ratio in comparison to **AmB** [66]. This study provides evidence that amide derivatives of **natamycin** are potential antifungal agents.

Rohira et al. synthesized an innovative cell-penetrating peptide (CPP) **natamycin** derivative for fungal keratitis management and evaluated its enhanced antifungal activity [67]. The MIC value of **Tat_2_natamycin** was 32 µg/mL when compared to **natamycin** (32 µg/mL) and **Tat_2_** (64 µg/mL). Furthermore, when different concentrations of **Tat_2_natamycin** and **Tat_2_** below their MIC values were tested for antifungal activity, the MIC of **Tat_2_** was found to be 48 µg/mL, whereas no change in MIC was observed for **Tat_2_natamycin**. The study concluded that CPP-conjugated **natamycin** (**Tat_2_natamycin**) had better tissue penetration and antifungal activity than the commercial formulation of natamycin. This could be attributed to higher bioavailability and the observed morphological changes on the fungal plasma membrane induced by the conjugate. Additionally, upon the conjugation of **natamycin** with **Tat_2_**, there was a significant reduction (~150-fold) in concentration from 50 mg/mL (concentration of marketed formulation) to 0.330 mg/mL (concentration of **Tat_2_natamycin** equivalent to 100 × 10^−6^ M) for effective antifungal activity against *Fusarium* sp. in vivo. These findings strongly suggest that **Tat_2_natamycin** is a promising peptide conjugate with a therapeutic effect at the micromolar range against fungal keratitis caused by *Fusarium* sp. [67].

## 4. Conclusions and Future Perspectives

Polyene derivatives have potent antifungal efficacy against most of the common fungal pathogens. Over the years, fungal pathogens have developed resistance to most antifungal drugs, thus limiting their efficacy in the treatment of fungal infections. Polyenes are mostly toxic to the host cell; hence, the development of polyene-based derivatives to reduce these toxic effects, overcome drug resistance, and enhance antifungal activity is crucial.

In this brief review, most of the derivatives exhibited superior or comparable antifungal activity compared to their corresponding reference molecules, indicating that the modification of the polyene structure greatly enhanced their antifungal properties. It has been observed that incorporating amide linkers at the carboxylic moiety of polyene molecules notably improves their antifungal properties, as evidenced by derivatives **4**, **5**, **6G**, and **18**. Considering the polyene derivatives synthesized to date, it is clear that further derivatives are necessary to thoroughly understand the SAR of these compounds. Although significant progress has been made, numerous research gaps remain. These include the synthesis of polyene-based hybrid compounds through hybridization with other bioactive compounds that possess known antifungal, bioavailability, and toxicological properties. Hybrid molecules, which combine two or more pharmacophores into a single compound, have emerged as a promising strategy in the search for new antifungal agents that can overcome the MDR. For instance, one such hybrid molecule (compound **17**) highlighted in this study incorporates gallic acid and has demonstrated enhanced antifungal activities. Additionally, there is a significant need for more in vivo evaluations of the synthesized polyene-based derivatives.

The exploration of synergistic interactions with existing antifungal agents may provide innovative strategies for combating multidrug-resistant pathogens. The exploration of nanoparticles [68,69,70] in combination with polyenes is an attractive approach for developing potent antifungal agents against co-infections. Continued research efforts aimed at elucidating their SAR, optimizing synthetic routes, and addressing challenges related to drug resistance and toxicity will be instrumental in realizing their therapeutic potential. Overall, polyene derivatives hold great promise as valuable drugs against infectious diseases.

## Figures and Tables

**Table 1:** MIC values of compound **6** and **6G** comparable to **AmB** [42].

Minimum Inhibitory Concentration (MIC, µg/mL)												
	*C. albicans* ATCC 24433		*C. parapsilosis* ATCC 22019		*C. krusei* 432M		*C. tropicalis* 3019		*C. glabrata* 61L			
Compound	24 h	48 h	24 h	48 h	24 h	48 h	24 h	48 h	24 h	48 h	*M. caris* B-200	*T. rubrum* 2002
**AmB**	0.125	0.25	0.25	0.5	0.25	0.5	0.06	0.25	0.05	0.25	0.25	0.25
**6** (amphamide)	0.03	0.06	0.03	0.06	0.125	0.25	0.03	0.06	0.03	0.06	0.5	0.5
**6G**	0.03	0.06	0.03	0.06	0.125	0.25	0.03	0.06	0.03	0.06	0.5	0.5

**Table 2:** MIC values of **AmB**, **BSA-AmB**, and **AmBisome** [51].

Antifungal Activity of AmB, BSA-AmB Conjugate, and AmBisome (MIC, µg/mL)			
Samples	*C. albicans*	*C. parapsilosis*	*C. neoformans*
**AmB**	0.53	0.4	0.78
**BSA-AmB**	0.87	0.67	1.1
**AmBisome**	078	1.5	0.78

**Table 3:** Antifungal activities of conjugates **7a**, **7b**, and **7c** [48].

Compounds	MIC (µg/mL)		
	*C. albicans*	*C. glabrata*	*C. neoformans*
**AmB**	0.5–1	0.5–1	0.25–0.5
**7a**	2–4	1–2	1–2
**7b**	4–8	2–4	2–4
**7c**	4–8	2–4	2–4

**Table 4:** Antifungal activity of compound **8** compared to that of **nystatin** and **AmB** [52].

	MIC (µg/mL)		
	Nystatin A1	AmB	Compound 8
*C. albicans* KCTC7965	4	1	2
*C. albicans* SC5314	4	1	4
*C. albicans* SL28	4	1	4
*C. albicans* SL38	4	1	2
*Cryptococcus humilicola* ATCC9949	-	0.5	1
*Saccharomyces cerevisiae* ATC9035	-	1	2
Hemolytic activity (MHC, µg/mL)	66.17 ± 0.90	4.65 ± 0.17	13.60 ± 0.19

**Table 5:** Antifungal activity of **AmB** nanoparticles [53].

Compounds	MIC (µg/mL)	
	*C. albicans*	*C. neoformans*
**ZnO**	>5	>5
**AmB**	0.1	0.05
**ZnO-AmB**	0.05	0.05
**ZnO-AmB-PEG**	0.00625	0.00625

**Table 6:** Antifungal activity of nystatin derivative compound **12** [58].

MIC (µg/mL)		
Compound	*C. albicans*	*A. niger*
**AmB**	5 ± 0.76	31.5 ± 2.1
**Econazole**	>35	>35
**Nystatin**	10 ± 2.7	22.5 ± 2.2
**12**	5 ± 1.2	23.6 ± 2.1
**Polymyxin B**	>35	>35

**Table 7:** Antifungal values of promising derivatives of **AmB** (**13**) and nystatin (**14**) [59].

MIC (µg/mL)								
Tested Strains	Compound							
	13a	14a	13b	14b	14c	14e	13	14
*C. parapsilosis* ATCC 22019	0.03/0.06	0.5/1	0.06/0.125	2/4	1/2	1/2	1/2	2/2
*C. albicans* ATCC10231	n/t	1/2	n/t	2/4	2/4	4/8	0.5/0.5	1/2
*A. fumigatus* ATCC46645	n/t	2	n/t	4	2	8	2	4
*A. niger* 37a	0.125	8	0.5	16	8	16	2	8
*T. rubrum* 2002	0.5	8	0.25	32	8	8	1	8
*C. krusei* 432M	0.25/0.5	4/4	0.25/0.5	8/8	16/16	8/16	0.5/1.0	4/4
*C. albicans* 604M	0.05/0.1	1/1	n/t	2/4	2/2	2/4	1/1	1/2
*C. albicans* 8R	0.05/0.1	1/2	n/t	4/4	2/4	2/4	1/1	1/2
*C. glabrata* 61L	0.03/0.06	1/1	n/t	2/2	1/2	2/2	1/2	1/2
*C. tropicalis* 3010	n/t	1/1	n/t	2/2	2/2	2/4	0.5/1	1/2
*C. parapsilosis* 58L	n/t	1/2	n/t	2/4	1/2	2/2	0.5/1	1/2

n/t = not tested.
